# *Ex vivo* expansion of circulating CD34^+^ cells enhances the regenerative effect on rat liver cirrhosis

**DOI:** 10.1038/mtm.2016.25

**Published:** 2016-04-27

**Authors:** Toru Nakamura, Hironori Koga, Hideki Iwamoto, Victor Tsutsumi, Yasuko Imamura, Masako Naitou, Atsutaka Masuda, Yu Ikezono, Mitsuhiko Abe, Fumitaka Wada, Takahiko Sakaue, Takato Ueno, Masaaki Ii, Cantas Alev, Atsuhiko Kawamoto, Takayuki Asahara, Takuji Torimura

**Affiliations:** 1 Division of Gastroenterology, Department of Medicine, Kurume University School of Medicine, Kurume, Japan; 2 Liver Cancer Division, Research Center for Innovative Cancer Therapy, Kurume University, Kurume, Japan; 3 Department of Infectomics and Molecular Pathogenesis, Center for Research and Advanced Studies, Mexico City, Mexico; 4 Group of Translational Stem Cell Research, Department of Pharmacology, Osaka Medical College, Takatsuki, Japan; 5 Department of Cell Growth and Differentiation, Center for iPS Cell Research and Application, Kyoto University, Kyoto, Japan; 6 Group of Vascular Regeneration Research, Institute of Biomedical Research and Innovation, Kyoto, Japan; 7 Department of Regenerative Medicine Science, Tokai University School of Medicine, Isehara, Japan

## Abstract

*Ex vivo* expansion of autologous cells is indispensable for cell transplantation therapy of patients with liver cirrhosis. The aim of this study was to investigate the efficacy of human *ex vivo*-expanded CD34^+^ cells for treatment of cirrhotic rat liver. Recipient rats were intraperitoneally injected with CCl_4_ twice weekly for 3 weeks before administration of CD34^+^ cells. CCl_4_ was then re-administered twice weekly for 3 more weeks, and the rats were sacrificed. Saline, nonexpanded or expanded CD34^+^ cells were injected via the spleen. After 7 days, CD34^+^ cells were effectively expanded in a serum-free culture medium. Expanded CD34^+^ cells were also increasingly positive for cell surface markers of VE-cadherin, VEGF receptor-2, and Tie-2. The expression of proangiogenic growth factors and adhesion molecules in expanded CD34^+^ cells increased compared with nonexpanded CD34^+^ cells. Expanded CD34^+^ cell transplantation reduced liver fibrosis, with a decrease of αSMA^+^ cells. Assessments of hepatocyte and sinusoidal endothelial cell proliferative activity indicated the superior potency of expanded CD34^+^ cells over non-expanded CD34^+^ cells. The inhibition of integrin αvβ3 and αvβ5 disturbed the engraftment of transplanted CD34^+^ cells and aggravated liver fibrosis. These findings suggest that expanded CD34^+^ cells enhanced the *preventive* efficacy of cell transplantation in a cirrhotic model.

## Introduction

In recent years, there have been several reports of the use of regenerative medicine techniques for treatment of liver disease using peripheral blood (PB) stem cells (*i.e.*, CD34^+^, CD133^+^)^1–5^ or bone marrow cells.^6–8^ Moreover, various basic studies using the cellular material responsible for the next generation of embryonic stem cells^9,10^ and induced pluripotent stem cells^11,12^ have been reported. Embryonic stem cells are associated with immunity problem; however, the problems of immune rejection are avoided when using autologous stem cells. There is a limit to the number of cells that can be harvested from a patient. We suggest using cells after amplification in culture as a remedy. In particular, a sufficient number of cells is essential for treatment for a wide range of organ damage, such as cirrhotic liver.

Asahara *et al*.^13^ reported that human PB-CD34^+^ cells contained a population enriched in endothelial progenitor cells (EPCs) that contributed to vasculogenesis as well as hematopoietic stem cells. Their involvement in neovascularization has been studied extensively in many fields including liver.^14–18^ We recently reported that transplantation of rat bone marrow-derived cultured EPCs in a rodent model of liver fibrosis markedly improved hepatic regeneration and promoted fibrolysis, thereby improving liver function and survival.^19–21^ Our findings suggested that the potential clinical value of cell therapy for the treatment of liver cirrhosis (LC) might be substantial. However, expanded EPCs, cultured with fetal bovine serum and bovine pituitary extracts, could not be directly used for clinical application because of a risk of zoonosis. In view of the clinical application, it may be required to establish an *ex vivo* amplification method for culturing under serum-free conditions. Masuda *et al*.^22^ has recently developed a serum-free culture system containing stem cell factor, thrombopoietin, vascular endothelial growth factor (VEGF), interleukin-6, and FMS-like tyrosine kinase-3 (Flt-3) ligand for colony-forming EPCs to enhance their regenerative potential. They reported that cultivation of umbilical cord blood (CB)-CD133^+^ cells for 7 days increased cell number and the frequency of definitive EPC colony development, resulting in an enhancement of vasculogenesis.

It is known that the number, proliferation, and function of EPCs decline in patients with aging, cardiovascular risk factors, or diabetes.^23–26^ Recently, we reported that autologous granulocyte-colony stimulating factor (G-CSF)-mobilized PB-CD34^+^ cell transplantation for patients with decompensated LC had therapeutic potential, but the colony-forming ability of patients with decompensated LC had been reduced.^5^ *Ex vivo* expansion of autologous cells is indispensable for cell transplantation therapy of patients with chronic disease including LC. Here, we examined whether the clinical-grade *ex vivo* expansion could restore the number and function of EPC in LC patients. We sought in this way to improve the transplantation of *ex vivo* expanded PB-CD34^+^ cells to LC patients. We tested the preventive efficacy of these expanded cells and nonexpanded PB-CD34^+^ cells in the treatment of carbon tetrachloride (CCl_4_)-induced cirrhotic liver.

## Results

### Characterization of *ex vivo* expanded G-CSF-mobilized PB-CD34^+^ cells

**Figure 1:**
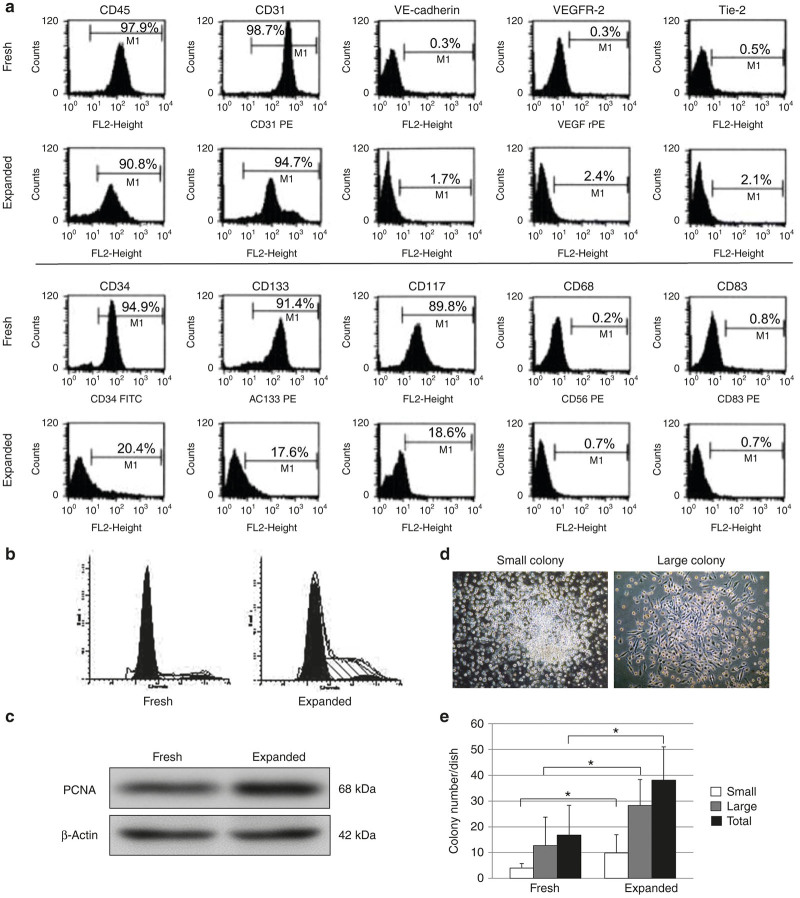
*Ex vivo* expanded G-CSF-mobilized PB-CD34^+^ cells restored vasculogenic potential of fresh PB-CD34^+^ cells. (**a**) PB-CD34^+^ cells were characterized by flow cytometric analysis. PB-CD34^+^ cells were also increasingly positive for cell surface markers of VE-cadherin, VEGFR-2, and Tie-2, whereas they were downregulated for CD34, CD133, and CD117 (*n* = 5). (**b**) Flow cytometric analysis of the cell cycle shows fresh and expanded PB-CD34^+^ cells. Expanded PB-CD34^+^ cells proliferated to an extent comparable with fresh PB-CD34^+^ cells. (**c**) Western blot analysis of the cell proliferation protein (PCNA) is shown. The expression level of PCNA was upregulated in expanded PB-CD34^+^ cells. (**d**) EPC colony-forming assay revealed two distinct colonies; primitive EPC-CFUs, and definitive EPC-CFUs. (**e**) After 20 days in culture, the number of EPC-CFUs per dish of expanded PB-CD34^+^ cells was significantly greater than that of fresh PB-CD34^+^ cells. *P < 0.05. Expanded, expanded PB-CD34^+^ cells; fresh, nonexpanded PB-CD34^+^ cells. PCNA, proliferating cell nuclear antigen.

After 7 days in culture, *ex vivo*-expanded G-CSF mobilized PB-CD34^+^ cells were effectively expanded (7.9 ± 8.1-fold). The expression of CD34 (20.4 ± 1.3%), CD133 (17.6 ± 0.9%), and CD117 (18.6 ± 1.1%) was downregulated in expanded PB-CD34^+^ cells (Figure 1a). Expanded PB-CD34^+^ cells were also characterized for expression of cell surface markers of CD45 (90.8 ± 0.2%), CD31 (94.7 ± 0.3%), VE-cadherin (1.7 ± 0.4%), VEGFR-2 (2.4 ± 0.2%), and Tie-2 (2.1 ± 0.3%), whereas they were negative for CD68 (0.7 ± 0.8%) and CD83 (0.7 ± 0.4%; Figure 1a).

Cell proliferation was analyzed using flow cytometry and western blotting. Expanded PB-CD34^+^ cells were compared with nonexpanded (fresh) PB-CD34^+^ cells. The percentage of the cell population in the G0/G1 phase in the fresh versus expanded PB-CD34^+^ cells was 79.8 versus 52.6%, 14.4 versus 42.4% in S phase, and 5.8 versus 5.0% in G2/M phase (Figure 1b). The expression level of proliferating cell nuclear antigen (PCNA) was upregulated in expanded PB-CD34^+^ cells (Figure 1c).

The primitive EPC-colony forming units (CFUs) and definitive EPC-CFUs were counted separately (Figure 1d). After 20 days in culture, the number of EPC-CFUs per dish of expanded PB-CD34^+^ cells was significantly greater than that of fresh PB-CD34^+^ cells (primitive EPC-CFUs: fresh, 4.0 ± 1.7; expanded, 9.8 ± 7.2; definitive EPC-CFUs: fresh, 12.7 ± 11.0; expanded, 28.3 ± 10.1; Figure 1e).

**Figure 2:**
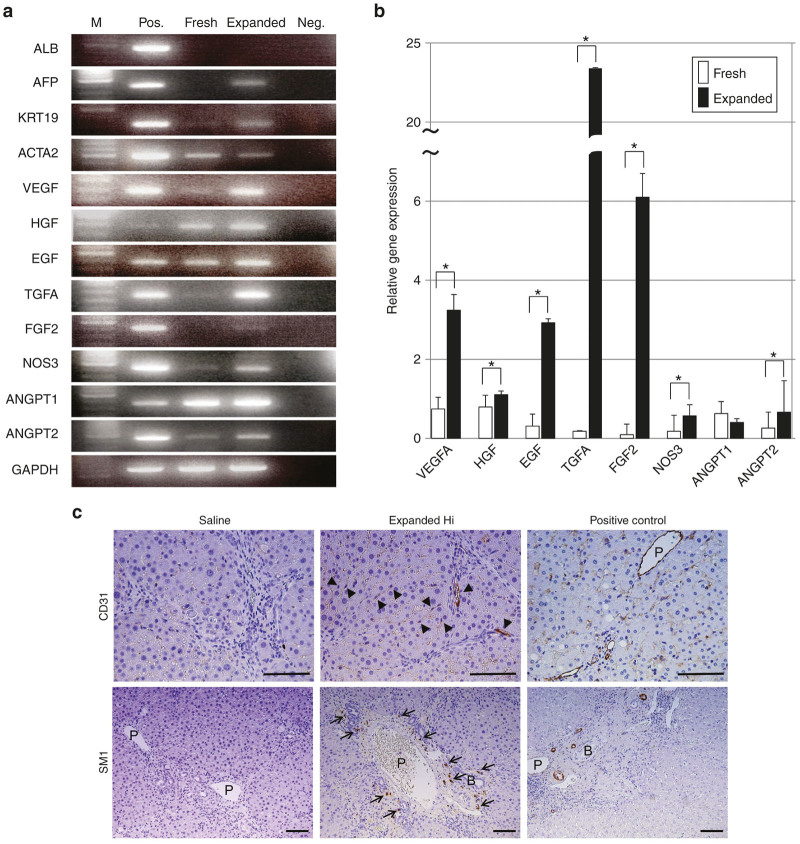
Characterization of *ex vivo* expanded G-CSF-mobilized PB-CD34^+^ cells *in vitro* and *in vivo*. (**a**) RT-PCR of expanded PB-CD34^+^ cells revealed human specific gene expression of *AFP*, *KRT19*, and *ACTA2*, but *ALB* was not observed. (**b**) The mRNA expression levels of *VEGFA*, *HGF*, *EGF*, *TGFA*, *FGF2*, *NOS3*, and *ANGPT2* in expanded PB-CD34^+^ cells were significantly higher than that in fresh PB-CD34^+^ cells by real-time PCR. (**c**) Distribution of transplanted expanded PB-CD34^+^ cells in CCl_4_-treated liver. At 3 weeks following transplantation, transplanted expanded PB-CD34^+^ cells stained positively for vascular and sinusoidal endothelial cells (staining for CD31) as well as vascular smooth muscle cells (staining for SM1). Bar = 100 µm. *P < 0.05. ACTA2, alpha2-smooth muscle actin; AFP, α-fetal protein; ANGPT, angiopoietin; EGF, epithelial growth factor; expanded, expanded PB-CD34^+^ cells; FGF, fibroblast growth factor; fresh, nonexpanded PB-CD34^+^ cells; GAPDH, glyceraldehyde-3-phosphate dehydrogenase; healthy, healthy individuals; HGF, hepatocyte growth factor; Hi, high-dose; LC, liver cirrhotic patients; M, molecular markers, Neg., negative control; NOS, nitric oxidase synthesis; Pos., positive control; TGF, transforming growth factor; VEGF, vascular endothelial growth factor.

The RT-PCR of expanded PB-CD34^+^ cells revealed the expression of human specific genes for *AFP*, *KRT19*, and *ACTA2*, but *ALB* was not detected (Figure 2a). To clarify the paracrine effects of transplanted cells, we measured the mRNA expression of various growth factors and proangiogenic factors in fresh and expanded PB-CD34^+^ cells using real-time PCR. The mRNA expression levels of *VEGFA*, *HGF*, EGF, *TGFA*, *FGF2*, *NOS3*, and *ANGPT2* in expanded PB-CD34^+^ cells were significantly higher than those in fresh PB-CD34^+^ cells (Figure 2a,b). In contrast, the expression level of *ANGPT1* in expanded PB-CD34^+^ cells was significantly lower than that in fresh PB-CD34^+^ cells (Figure 2b).

### Transplanted expanded PB-CD34^+^ cells differentiated into vascular and sinusoidal endothelial cells and vascular smooth muscle cells

Human CD31-positive endothelial cells derived from transplanted expanded PB-CD34^+^ cells were located near the vessels within the fibrous septa and along the hepatic sinusoids of CCl_4_-treated livers (Figure 2c). Moreover, we observed human SM1-positive vascular smooth muscle cells. Human vascular smooth muscle cells derived from expanded PB-CD34^+^ cells were located in the vasculature within the periportal areas (Figure 2c). However, the transplanted expanded PB-CD34^+^ cells did not differentiate into human keratin19-positive bile ductular epithelial cells, human albumin-positive hepatocytes, or human AFP-positive cells (data not shown). We did not detect any human cells in saline-infused livers treated with CCl_4_ (Figure 2c).

### Transplantation of expanded PB-CD34^+^ cells prevented the progression of liver fibrosis in a dose-dependent manner

**Figure 3:**
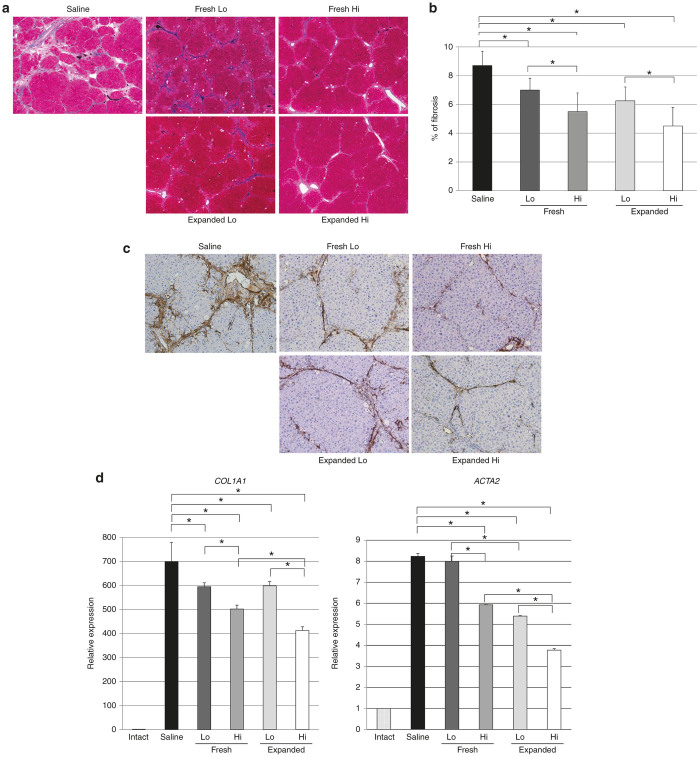
Transplantation of expanded PB-CD34^+^ cells prevented the progression of liver fibrosis in a dose-dependent manner. (**a**) Fibrosis was less notable in expanded PB-CD34^+^ cell-transplanted livers than in saline-infused livers according to Mallory’s Azan staining. Original magnification: ×40. (**b**) Semi-quantitative analysis indicated that the relative extents of the fibrotic area were prevented significantly in a dose-dependent manner in rats receiving fresh and expanded PB-CD34^+^ cell transplantation after 6 weeks of CCl_4_ treatment (*n* = 8). (**c**) Both fresh and expanded PB-CD34^+^ cell-transplanted livers had fewer αSMA-positive cells than did livers receiving saline. Original magnification: ×100. (**d**) Quantitative real-time PCR of mRNA expression levels of *COL1A1* and *ACTA2* after treatment with PB-CD34^+^ cells or saline. The expression of *COL1A2* and *ACTA2* mRNAs after 6 weeks of CCl_4_ treatment was decreased in a dose-dependent manner in fresh and expanded PB-CD34^+^ cell-transplanted livers (*n* = 8). *P < 0.05. Expanded, expanded PB-CD34^+^ cell-transplanted livers; fresh, freshly isolated PB-CD34^+^ cell-transplanted livers; Hi, high-dose cell-transplanted livers; Lo, low-dose cell-transplanted livers; intact, normal livers; saline: saline-infused livers.

Reduction of liver fibrosis by transplantation of expanded PB-CD34^+^ cells was demonstrated by Mallory’s Azan histologic staining (Figure 3a) and by immunohistochemical analysis for αSMA (Figure 3c) in CCl_4_-treated livers. Semi-quantitative analysis indicated that the relative extent of the fibrotic area was significantly reduced in a dose-dependent manner for transplanted fresh PB-CD34^+^ cells and expanded PB-CD34^+^ cells (saline, 8.7 ± 1.0%; fresh low-dose (Lo) group, 7.0 ± 0.8%; fresh high-dose (Hi) group, 5.5 ± 1.3%; expanded Lo group, 6.3 ± 1.0%; expanded Hi group, 4.5 ± 1.3%; Figure 3b). However, there was no significant difference in liver fibrosis between fresh PB-CD34^+^ cell transplantation and expanded PB-CD34^+^ cell transplantation. The number of αSMA-positive cells in the liver transplanted with fresh or expanded PB-CD34^+^ cells was fewer than that in nontreated liver (Figure 3c). These inhibitory effects were observed ubiquitously throughout the liver. Real-time PCR showed that the expression of *COL1A1*, and *ACTA2* mRNAs was significantly decreased in a dose-dependent manner in fresh and expanded PB-CD34^+^ cell-transplanted livers compared to nontreated livers with the exception of fresh Lo PB-CD34^+^ cell-transplanted livers (Figure 3d).

### Expanded PB-CD34^+^ cells secrete MMPs

**Figure 4:**
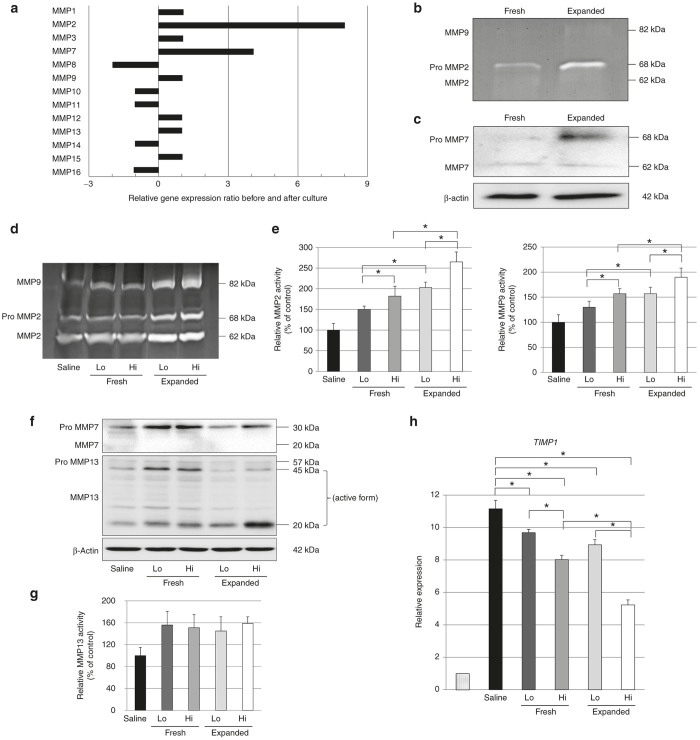
Expanded PB-CD34^+^ cells secrete MMPs. (**a**) The RT^2^ Profiler PCR Array analysis against the extracellular matrix revealed that the mRNA levels of *MMP2* and *MMP7* expression were upregulated in expanded PB-CD34^+^ cells compared with fresh PB-CD34^+^ cells. (**b**) Gelatin zymography showed that only the pro-form of MMP2 was upregulated in expanded PB-CD34^+^ cells. (**c**) Western blot showed that only the pro-form of MMP7 was upregulated in expanded PB-CD34^+^ cells. (**d** and **e**) *In vivo* results, gelatin zymography indicated that the active forms of MMP2 and MMP9 were elevated in a dose-dependent manner in livers transplanted with fresh and expanded PB-CD34^+^ cells after 6 weeks of CCl_4_ treatment (*n* = 8). (F, G) Only the pro-form of MMP7 was detected in livers after 6 wk of CCl_4_ treatment. The active form of MMP13 was significantly upregulated in the livers of fresh and expanded PB-CD34^+^ cell-transplanted livers compared to saline-infused livers, although a nonexpanded/expanded effect was observed and no significant dose-response relationship was observed (*n* = 8). (**h**) Quantitative real-time PCR of mRNA expression levels of TIMP1 after treatment with PB-CD34^+^ cells or saline. The expression of TIMP1 mRNAs after 6 weeks of CCl_4_ treatment was decreased in a dose-dependent manner in fresh and expanded PB-CD34^+^ cell-transplanted livers (*n* = 8). *P < 0.05. Expanded, expanded PB-CD34^+^ cell-transplanted livers; fresh, freshly isolated PB-CD34^+^ cell-transplanted livers; Hi, high-dose cell-transplanted livers; Lo, low-dose cell-transplanted livers; Saline: saline-infused livers.

The RT^2^ Profiler PCR Array analysis for extracellular matrix revealed that the mRNA levels of *MMP2* and *MMP7* expression were upregulated in expanded PB-CD34^+^ cells compared with fresh PB-CD34^+^ cells (Figure 4a). However, only the proforms of MMP2 and MMP7 proteins were upregulated in expanded PB-CD34^+^ cells (Figure 4b,c).

*In vivo*, gelatin zymography and western blot analysis demonstrated that the active form of MMP2 and MMP9 was elevated in a dose-dependent manner in livers transplanted with fresh and expanded PB-CD34^+^ cells (Figure 4d,e). The only pro-form of MMP7 was detected in the livers (Figure 4f). The active form of MMP13 was significantly upregulated in the livers transplanted with fresh and expanded PB-CD34^+^ cells compared to nontreated livers, although no significant dose–response relationship was observed. There was also no difference of MMP13 expression between fresh and expanded PB-CD34+ cell transplanted livers. (Figure 4f,g). We also measured the expression of tissue inhibitor of metalloproteinase (TIMPs) by real-time PCR. The expression levels of TIMP1 mRNAs was significantly decreased in a dose-dependent manner in livers transplanted with fresh and expanded PB-CD34^+^ cells (Figure 4h).

### Transplantation of expanded PB-CD34^+^ cells accelerated hepatic regeneration

**Figure 5:**
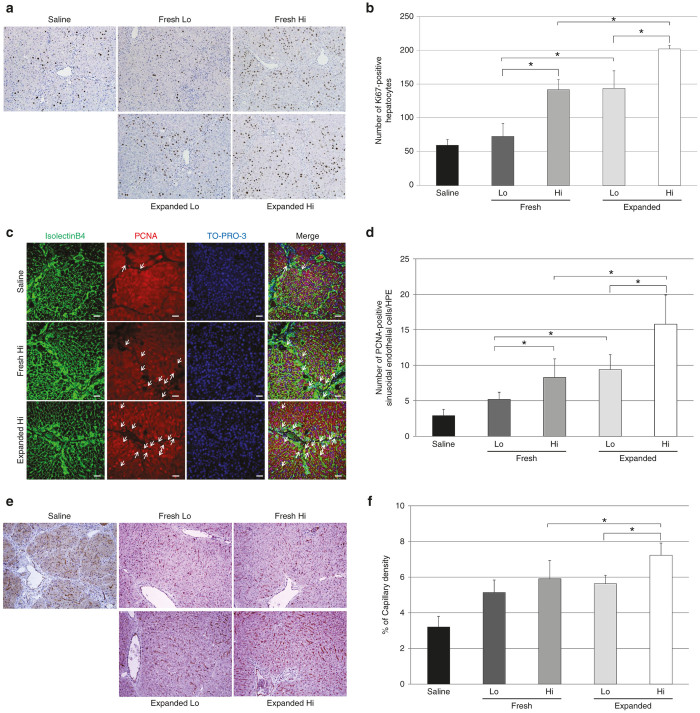
Transplantation of expanded PB-CD34^+^ cells accelerated hepatic regeneration and reconstituted sinusoidal blood vessels. (**a** and **b**) Immunohistochemial analysis of hepatocyte proliferation (staining for Ki67) in livers treated with CCl_4_. The Ki67 labeling index for hepatocytes in livers transplanted with expanded PB-CD34^+^ cells was significantly higher than that in livers transplanted with fresh PB-CD34^+^ cells (*n* = 8). Original magnification: ×100. (**c** and **d**) Immunohistochemial analysis of sinusoidal endothelial cell proliferation (staining for PCNA) in livers after 6 weeks of CCl_4_ administration. The number of PCNA^+^ (red) isolectin-B4^+^ (green) cells in livers transplanted with expanded PB-CD34^+^ cells was significantly higher than that in livers transplanted with fresh PB-CD34^+^ cells. Arrows indicate PCNA^+^ isolectin-B4^+^ cells (*n* = 8). Scale bar = 50 µm. (**e**) NOS3 expression in livers after 6 weeks of CCl_4_ administration. Both fresh and expanded PB-CD34^+^ cell-transplanted livers had increased vascular density compared with saline-infused livers. Original magnification: ×100. (**f**) The percentage of capillary density in livers transplanted with a Hi dose of expanded PB-CD34^+^ cells was significantly higher than that in livers transplanted with a Hi dose of fresh PB-CD34^+^ cells (*n* = 8). Original magnification: ×100. *P < 0.05. Expanded, expanded PB-CD34^+^ cell-transplanted livers; fresh, freshly isolated PB-CD34^+^ cell-transplanted livers; Hi, high-dose cell-transplanted livers; Lo, low-dose cell-transplanted livers; PCNA, proliferating cell nuclear antigen; Saline: saline-infused livers.

The Ki67 labeling index for hepatocytes was increased in a dose-dependent manner for transplanted PB-CD34^+^ cells. Furthermore, the Ki67 labeling index for hepatocytes in the livers transplanted with expanded PB-CD34^+^ cells was significantly higher than that in the livers transplanted with fresh PB-CD34^+^ cells. (saline, 59.7 ± 8.3; fresh Lo group, 72.8 ± 19.2; fresh Hi group, 141.7 ± 15.2; expanded Lo group, 143.3 ± 26.3; expanded Hi group, 202.0 ± 5.1; Figure 5a,b). In addition, to evaluate whether expanded PB-CD34^+^ cell transplantation promoted the proliferation of endothelial cells, we performed double immunohistochemistry. The number of cells double positive for PCNA and isolectin-B4 was increased in a dose-dependent manner for the number of transplanted PB-CD34^+^ cells. Furthermore, the number of PCNA^+^ isolectin-B4^+^ cells in the liver transplanted with expanded PB-CD34^+^ cells was significantly higher than that in the liver transplanted with fresh PB-CD34^+^ cells. (saline, 2.9 ± 0.9; fresh Lo group, 5.2 ± 1.0; fresh Hi group, 8.3 ± 2.6; expanded Lo group, 9.4 ± 2.1; expanded Hi group, 15.8 ± 4.1; Figure 5c,d).

### Transplantation of expanded PB-CD34^+^ cells promotes hepatic angiogenesis

The expression of NOS3 protein was seen in hepatic sinusoidal endothelial cells and vascular endothelial cells. The livers transplanted with fresh or expanded PB-CD34^+^ cells resulted in the promotion of vessel formation. In addition, liver sections with fresh or expanded PB-CD34^+^ cell transplantation showed a continuous staining pattern along the sinusoid (Figure 5e). Semi-quantitative analysis demonstrated that the NOS3-positive area in the Hi group of expanded PB-CD34^+^ cell-transplanted livers had markedly and significantly increased vascular density compared with the Hi group of fresh PB-CD34^+^ cell-transplanted livers (saline, 3.2 ± 0.7; fresh Lo group, 5.1 ± 1.0; fresh Hi group, 5.9 ± 0.5; expanded Lo group, 5.6 ± 0.7; expanded Hi group, 7.2 ± 0.2; Figure 5f).

### Inhibition of integrin (ITG) αvβ3 and αvβ5 disturbed the engraftment of transplanted expanded PB-CD34^+^ cells and aggravated liver fibrosis

**Figure 6:**
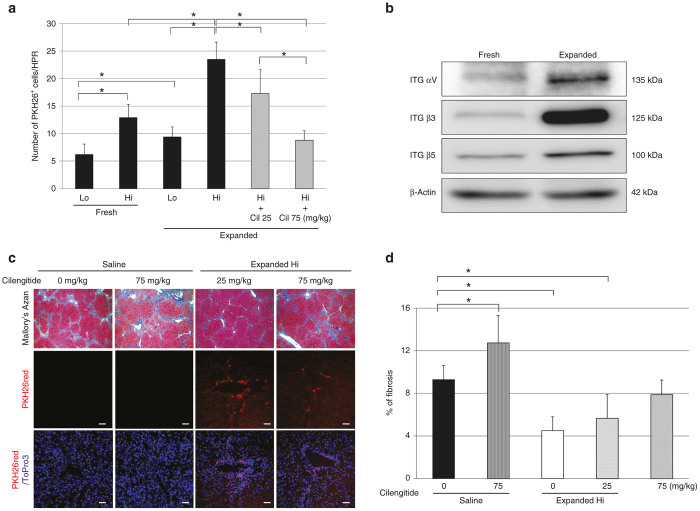
Inhibition of ITG αvβ3 and αvβ5 disturbed the engraftment of transplanted expanded PB-CD34^+^ cells and aggravated liver fibrosis. (**a**) The number of PKH26 red^+^ transplanted PB-CD34^+^ cells per high-power field. Transplanted PB-CD34^+^ cells migrated in a dose-dependent manner into the liver. The migration of expanded PB-CD34^+^ cells was significantly upregulated compared with that of fresh PB-CD34^+^ cells (*n* = 8). (**b**) The expression of integrin αv, β3 and β5 by western blotting. (**c** and **d**) Treatment with cilengitide suppressed the migration of transplanted expanded PB-CD34^+^ cells into the liver and showed an increase of liver fibrosis in a dose-dependent manner (*n* = 5). Original magnification upper: ×40; bar = 50 µm. *P < 0.05. Cil, cilengitide; Expanded, expanded PB-CD34^+^ cell-transplanted livers; Fresh, freshly isolated PB-CD34^+^ cell-transplanted livers; Hi, high-dose cell-transplanted livers; ITG, integrin; LC, LC patient-derived PB-CD34^+^ cells; Lo, low-dose cell-transplanted livers; Saline: saline-infused livers.

Transplanted PB-CD34^+^ cells migrated in a dose-dependent manner into the liver. The migration of expanded PB-CD34^+^ cells was significantly upregulated compared with that of fresh PB-CD34^+^ cells (fresh Lo group, 6.2 ± 1.9; fresh Hi group, 12.9 ± 2.4; expanded Lo group, 9.4 ± 1.8; expanded Hi group, 23.5 ± 3.1; Figure 6a).

The RT^2^ Profiler PCR Array analysis for adhesion molecules showed that the expression of ITG β3 was the most upregulated gene in expanded PB-CD34^+^ cells compared with fresh PB-CD34^+^ cells. ITG αv that form heterodimers with ITG β3 and β5 was also increased. Western blot analysis clarified that ITG αv, β3, and β5 expression was greatly upregulated in expanded PB-CD34^+^ cells compared with fresh PB-CD34^+^ cells (Table 1 and Figure 6b).

Cilengitide is an antagonist selective for ITG αvβ3 and αvβ5. Treatment with cilengitide suppressed the migration of transplanted expanded PB-CD34^+^ cells into the liver (expanded Hi without cilengitide, 23.5 ± 3.1; expanded Hi with 25 mg/kg cilengitide, 17.3 ± 4.2; expanded Hi with 75 mg/kg cilengitide, 8.8 ± 1.7; Figure 6a) and increase of liver fibrosis in a dose-dependent manner (saline without cilengitide, 9.3 ± 1.4%; saline with 75 mg/kg cilengitide, 12.7 ± 2.6%; expanded Hi without cilengitide, 4.5 ± 1.3%; expanded Hi with 25 mg/kg cilengitide, 5.6 ± 2.2%; expanded Hi with 75 mg/kg cilengitide, 7.9 ± 1.3%; Figure 6c,d).

## Discussion

We reported that the transplantation of nonexpanded human PB-CD34^+^ cells ameliorated established liver fibrosis, improved liver function, and the survival rate of CCl_4_-induced liver fibrosis in the nude rat model.^27^ Transplanted PB-CD34^+^ cells promoted fibrolysis and regeneration of fibrotic liver. Based on our basic research, we started a clinical trial of G-CSF-mobilized nonexpanded PB-CD34^+^ cell transplantation in patients with decompensated LC, and we demonstrated that PB-CD34^+^ cell therapy is feasible, safe, and effective in slowing the decline of hepatic reserve function.^5^ Through this clinical study, we discovered that the cell number and the colony-forming ability of PB-CD34^+^ cells in patients with decompensated LC were reduced.

In our study, G-CSF-mobilized PB-CD34^+^ cells were used as an EPC-enriched population based on the study reported by Asahata *et al*.^13^ These cells were expanded using a serum-free quality and quantity control culture system recently developed by Masuda *et al*.^22^ This culture system is a functional culture system that not only increases the cell numbers of EPCs but also increases the frequency in definitive EPC colony development, resulting in an enhancement of vasculogenesis. Our *in vitro* experiments demonstrated that LC patient-derived expanded PB-CD34^+^ cells were increased in cell number, definitive colony formation, and the expression of proangiogenic growth factors compared with fresh PB-CD34^+^ cells. In addition, production of proangiogenic growth factors also enhanced the proliferation of expanded PB-CD34^+^ cells compared with fresh PB-CD34^+^ cells.

Next, we investigated the antifibrotic effect of expanded PB-CD34^+^ cell transplantation and fresh PB-CD34^+^ cell transplantation. The progression of liver fibrosis was reduced both in fresh and in expanded PB-CD34^+^ cell-transplanted rats in a dose-dependent manner of transplanted cells. However, there was no significant difference in improvement of liver fibrosis between fresh PB-CD34^+^ cell-transplanted and expanded PB-CD34^+^ cell-transplanted livers. Liver fibrosis is the result of an imbalance between production and dissolution of extracellular matrix (ECM). In our results, mRNA levels of *COL1A1* and *ACTA2*, markers of activated hepatic stellate cells, were significantly decreased in expanded PB-CD34^+^ cell-transplanted livers compared with fresh PB-CD34^+^ cell-transplanted livers. However, no relationship and no nonexpanded/expanded effect were observed. Interstitial collagenase (*i.e.*, MMP13) is an important enzyme for collagenolysis in liver fibrosis and type-1 collagen, that increased predominantly in liver fibrosis, can be mainly degraded by MMP13.^28^ In the present study, the active form of MMP13 was upregulated in the livers of fresh and expanded PB-CD34^+^ cell-transplanted livers compared to nontreated livers, although a nonexpanded/expanded effect was observed and no significant dose–response relationship was observed. In addition, there was no difference in the expression levels of MMPs between before and after cell culture. From these results, we believe that there was no significant difference in the improvement of liver fibrosis between fresh PB-CD34^+^ cell-transplanted livers and expanded PB-CD34^+^ cell-transplanted livers.

We found a significant proliferation of hepatocytes and sinusoidal endothelial cells after transplantation of expanded PB-CD34^+^ cells. The possible explanation for superior hepatic regeneration is the upregulation of growth factor secretion for autocrine and paracrine action after PB-CD34^+^ cell transplantation. Our previous studies demonstrated that rat EPCs and human PB-CD34^+^ cells expressed multiple proangiogenic growth factors and the transplantation of these cells contributed to the hepatic regeneration.^19,27^ Masuda *et al*.^22^ showed that the gene expression of *VEGF* and *HGF* in expanded umbilical CB-CD133^+^ cells was upregulated compared with nonexpanded umbilical CB-CD133^+^ cells. Using real-time PCR, we confirmed that gene expression of many proangiogenic growth factors in expanded PB-CD34^+^ cells was significantly upregulated compared with fresh PB-CD34^+^ cells. These data suggest that expanded PB-CD34^+^ cells became an optimal cell source for preventive application.

We noticed that transplanted PB-CD34^+^ cells engrafted in a dose-dependent manner, and the migration of expanded PB-CD34^+^ cells was significantly increased compared with that of fresh PB-CD34^+^ cells. We hypothesized that some adhesion molecules might be upregulated in the process of cell expansion. The RT^2^ Profiler PCR Array analysis indicated that the levels of the ITG family, especially ITG αv and β3 expression were markedly upregulated in expanded PB-CD34^+^ cells compared with fresh PB-CD34^+^ cells. ITG αvβ3 interacts with several ECM proteins including vitronectin and fibronectin.^29^ ITG αvβ3 is expressed mainly on endothelial cells, and it plays an important role in endothelial cell migration.^30^ We investigated whether pharmacological inhibition of ITG αvβ3 and αvβ5 could block the engraftment of the expanded PB-CD34^+^ cells and worsen rat liver fibrosis. To block ITG αvβ3 and αvβ5 action in a specific manner, we used cilengitide, which is an antagonist for ITG αvβ3 and αvβ5.^31,32^ As cilengitide inhibits ITG αvβ3 and αvβ5 from human, bovine, and rat origin, it can be appropriately used in both experimental and clinical studies.^33,34^ Indeed, 3 weeks of treatment with cilengitide resulted in an increase of liver fibrosis compared with the untreated saline groups by Mallory’s Azan staining. Moreover, treatment with cilengitide resulted in prevention of the engraftment of the expanded PB-CD34^+^ cells and worsened liver fibrosis in a dose-dependent manner. The specific inhibition of ITG αvβ3 and αvβ5 by cilengitide experimentally decreased angiogenesis but worsened experimental liver fibrosis.^34^ These data support a direct role of transplanted expanded PB-CD34^+^ cells in hepatic regeneration.

In conclusion, we demonstrated that LC patient-derived PB-CD34^+^ cells were expanded about eightfold and PB-CD34^+^ cells expansion increased the expression of proangiogenic growth factors and adhesion molecules. We also clearly demonstrated that the serum-free expanded PB-CD34^+^ cells transplanted into experimental liver fibrotic nude rats accelerated hepatic regeneration. Because the expanded PB-CD34^+^ cells highly expressed ITG αvβ3 and αvβ5 proteins, the migration, and adhesion ability was enhanced and the expanded PB-CD34^+^ cells were easy to engraft in fibrotic liver tissues. In addition, the transplanted expanded PB-CD34^+^ cells supplied proangiogenic growth factors around liver tissues and promoted hepatic angiogenesis. Thus, we believe that patients will benefit from infusion of cells that have been exposed to *ex vivo* expansion and differentiation conditions compared to fresh, nonexpanded, undifferentiated cells.

## Materials and Methods

### Animals

Male 5-week-old athymic nude rats (F344/N Jcl rmu/rmu, CLEA Japan, Shizuoka, Japan) were used in this study. Animals were maintained in temperature-controlled rooms (21 ± 2 °C) under a 12/12 hour dark/light cycle and allowed food (standard laboratory chow) and water *ad libitum*. The experimental protocol was approved by the Ethics Review Committee for Animal Experimentation of Kurume University School of Medicine.

### Collection and isolation of CD34^+^ cells

PB total mononuclear cells were obtained from a cirrhotic Japanese male patient. This patient gave informed written consent to participate. The study protocol conformed to the ethical guidelines of the 1975 Declaration of Helsinki as reflected in prior approval by the institutional ethics committee of the Kurume University School of Medicine. The patient received a daily subcutaneous injection of G-CSF (Kyowa Hakko Kirin, Tokyo, Japan), 10 µg/kg/day for 5 days, to increase the number of PB-CD34^+^ cells. PB-CD34^+^ cells were isolated from total mononuclear cells with a magnetic cell sorting system, CliniMACS (Miltenyi Biotec, Auburn, CA).^5^ The CD34^+^ cell fraction had a purity of >99%, as determined by flow cytometric analysis of cells labeled with a CD34-specific monoclonal antibody (Becton-Dickinson, San Jose, CA).^5^

### *Ex vivo* expansion of CD34^+^ cells

At first, 50,000 PB-CD34^+^ cells in 2 ml of medium were plated into each well of a six-well tissue culture dish (Primaria; BD Biosciences, San Diego, CA) and cultured in a suspension manner using serum-free culture medium (StemSpam SFEM; StemCell Technologies, Vancouver, Canada) containing 50 ng/ml VEGF (Wako, Richmond, VA), 100 ng/ml stem cell factor (Wako), 20 ng/ml interleukin-6 (Wako), 100 ng/ml Flt-3 ligand (Wako), and 20 ng/ml thrombopoietin (Wako) for 7 days, as described previously.^22,35–37^

### Cell labeling (PKH26)

Experiments were carried out to detect transplanted cells as follows: the cells were labeled with red fluorescent marker PKH26-red (Sigma Chemical, St Louis, MO) following the manufacturer’s instructions before CD34^+^ cell transplantation into rats, as described previously.^19–21^

### EPC-colony forming unit (CFU) assay

To investigate the vasculogenic potential of nonexpanded and expanded CD34^+^ cells, we performed the EPC-CFU assay using semi-solid culture medium (MethoCult SF; StemCell Technologies) with proangiogenic growth factors in 35-mm Primaria dishes (BD Biosciences), as described previously.^5,22,37–40^ Twenty days after initiation of the culture, the number of adherent colonies per dish was measured using a gridded scoring dish (StemCell Technologies) under light microscopy. The primitive EPC-CFUs (small cells) and definitive EPC-CFUs (large cells) were counted separately.^5,22,37–40^

### Flow cytometry and cell cycle analysis

Human nonexpanded and expanded PB-CD34^+^ cells (*n* = 5) were analyzed by flow cytometry (FACSCalibur flow cytometer, (Becton-Dickinson (BD), San Jose, CA). Dead cells were excluded by propidium iodide (PI) staining (Sigma, St Louis, MO). The CD34^+^ cells were incubated with a FcR blocking reagent (Miltenyi Biotec, Auburn, CA) and incubated with the monoclonal antibodies for 30 minutes at 4 °C. The stained cells were washed, resuspended, and then analyzed using Quad Statistics of CellQuest software (BD). The following monoclonal antihuman antibodies were used to characterized the CD34^+^ cell population: CD34-FITC (BD), CD31-PE (BD), CD133-PE (Miltenyi Biotec, Auburn, CA), CD68-PE (BD), CD83-PE (BD), VE-cadherin-PE (BD), VEGFR-2-PE (R&D Systems, Minneapolis, MN), Tie-2 (BD), CD117-PE (BD), CD45-PE (BD), IgG2a-FITC isotope controls (Miltenyi Biotec), and IgG1-PE isotope controls (Miltenyi Biotec).^27^

The DNA content analysis was assessed by staining ethanol-fixed cells with PI and monitoring with the FACSCalibur flow cytometer. At least 20,000 cells were collected and analyzed with CellQuest software. Cell cycle distributions were calculated with ModFit LT cell-cycle analysis software (Verity Software House, Topsham, ME).

### Experimental conditions and transplantation of CD34^+^ cells

Liver fibrosis was induced by carbon tetrachloride (CCl_4_; Wako, Osaka, Japan). The nude rats received i.p. injections of 50% CCl_4_ (10 mg/kg body weight) twice weekly for 3 weeks. After CCl_4_ treatment, the nude rats underwent transplantation of 5 × 10^4^ (low-dose group, Lo) or 1 × 10^6^ (high-dose group, Hi) nonexpanded or expanded PB-CD34^+^ cells/kg in 150 µl of saline or saline solely via spleen (*n* = 8 in each group). Following PB-CD34^+^ cell transplantation, CCl_4_ administration was continued twice weekly for another 3 weeks. The rats were sacrificed after this 6-week period of CCl_4_ injection.

We investigated whether inhibition of ITG αvβ3 and αvβ5 could block the engraftment of the expanded PB-CD34^+^ cells. To block ITG αvβ3 and αvβ5, which specially inhibits ITG αvβ3 and αvβ5 signaling, we used cilengitide (EMD121974; Merck, Darmstadt, Germany).^31,32^ The rats were divided into 4 groups: (i) rats underwent transplantation with 150 µl of saline solely, (ii) rats underwent transplantation of 150 µl of saline with Hi cilengitide (75 mg/kg), (iii) rats underwent transplantation of 1 × 10^6^ expanded PB-CD34^+^ cells/kg in 150 µl of saline with high-dose cilengitide, (iv) rats underwent transplantation of 1 × 10^6^ expanded PB-CD34^+^ cells/kg in 150 µl of saline with Lo cilengitide (25 mg/kg) via spleen (*n* = 5 in each group). Cilengitide was administered intraperitoneally twice weekly between 22 and 42 days after CCl_4_ administration.^41^ The rats were sacrificed after this 6-week period of CCl_4_ injection.

### Total RNA extraction, cDNA synthesis, and reverse-transcription RT-PCR

Total RNA was isolated using Isogen kit (Nippon Gene, Tokyo, Japan) according to the manufacturer’s instructions. The high-capacity RNA-to-cDNA kit protocol (Applied Biosystems, Foster City, CA) was followed by transcription of 1 μg total RNA into single-stranded cDNA. The resulting cDNA then was amplified by PCR with human-specific primer pairs for albumin (ALB), alpha-fetal protein (AFP), keratin19 (KRT19), α2-smooth muscle actin (ACTA2), VEGF, hepatocyte growth factor (HGF), epithelial growth factor (EGF), transforming growth factor-α (TGFA), fibroblast growth factor-2 (FGF2), nitric oxidase synthesis 3 (NOS3) angiopoietin (ANGPT)-1, ANGPT2, and glyceraldehyde-3-phosphate dehydrogenase (*GAPDH*; CD31, CD34, *ALB*, *AFP*, *KRT19*, and *ACTA2*, 40 cycles; *HGF*, *TGFA*, *EGF*, *VEGF*, and *FGF2*, 35 cycles; and *GAPDH*, 28 cycles). PCR products were resolved in 1.5% agarose gels and visualized by ethidium bromide staining and ultraviolet illumination. The amplification was performed on a an Applied Biosystems Model No.9902 Veriti Thermal Cycler (Applied Biosystems) with the following cycling profile: denaturation at 94 °C for 30 seconds, annealing (see in Table 2) for 30 seconds, and extension at 72 °C for 1 minutes. Primer pair sequences are presented in Table 2.^27^

### TaqMan real-time quantitative PCR analysis

One microgram of total RNA was reverse transcribed by the TaqMan Reverse Transcription Reagent kit (Applied Biosystems) according to the manufacturer’s instructions. The following TaqMan Gene Expression Assays were purchased (Applied Biosystems): *COL1A1 (Assay ID Rn01463848_m1)*, *ACTA2* (Assay ID Rn01759928_g1), *TIMP1 (Assay ID Rn01430873_g1), VEGFA* (Assay ID Hs00900055_m1), *HGF* (Assay ID Hs00300159_m1), *EGF* (Assay ID Hs01099999_m1), *TGFA* (Assay ID Hs00608187_m1), *FGF2* (Assay ID Hs00266645_m1), *NOS3* (Assay ID Hs01574659_m1), *ANGPT1* (Assay ID Hs00375822_m1), *ANGPT2* (Assay ID Hs01048042_m1) and *B2M* (Assay ID Hs00984230_m1). The cycling parameters were initiated by 10 min at 95 °C, followed by 40 cycles of 15 seconds at 95 °C and 40 sec at 60 °C using StepOne Plus Real-Time PCR System (Applied Biosystems). Amplification reactions were performed in triplicate, and DNA contamination controls were included. Relative quantification of gene expression was performed according to the ΔΔ-CT method using StepOne Software 2.0 (Applied Biosystems), and B2M was used as a reference for the normalization of the results.

To analyze the signaling pathway under extracellular matrix and adhesion molecules, the RT^2^ Profiler PCR Array System (QIAGEN, Valencia, CA) was used. According to the manufacturer’s protocol, 40 μl cDNA was mixed with 2× SABiosciences RT^2^ qPCR Master Mix (QIAGEN) and H_2_O to a total volume of 2,700 μl. Subsequently, 25 μl of the mixture was placed into each well of the PCR array. Thermal cycling recommended by plates manufacturer for StepOne Plus Real-Time PCR System was used. To analyze the expression of genes, RT^2^ Profiler PCR Array Data Analysis Suite was used.

### Immunohistochemical analysis

Liver tissues were fixed in buffered formalin, embedded in paraffin, sectioned and mounted on silanized slides (Dakocytomation, Kyoto, Japan). Tissue sections were either subjected to Mallory’s Azan staining or were analyzed histochemically using antibodies against α-smooth muscle actin (αSMA; DAKO, A/S, Denmark), NOS3 (Transduction Laboratories, Lexington, KY), and Ki67 (DAKO, Kyoto, Japan). Immunoreactivity was visualized using an EnVision^+^ system (DAKO North America, Carpinteria, CA) and diaminobenzidine commercial kit (Liquid DAB^+^ Substrate Chromogen System; DakoCytomation California, Carpinteria, CA). The tissues in the blue-stained area in the Mallory’s Azan and NOS3-positive area were calculated using computer-assisted image software (WinROOF, Mitani, Tokyo, Japan) by a technician who was blinded to the treatment regimens (under ×40 magnification, five fields for each of eight rats).

To detect transplanted human cells in rat fibrotic liver, immunohistochemistry was performed with the following human-specific antibodies: human-specific CD31 (DAKO, Glostrup, Denmark) to detect human endothelial cells; human-specific keratin19 (DAKO, A/S, Denmark) to detect human ductal epithelial cells; human-specific smooth muscle myosin heavy chain-1 (SM1; Yamasa Shoyu, Choshi, Chiba, Japan) to detect human vascular smooth muscle cells; human-specific albumin (DAKO, A/S, Denmark) to detect human hepatocytes, and AFP (DAKO, Kyoto, Japan). We confirmed that the antihuman antibodies did not cross react with rat tissues by histochemical staining for CD31, keratin19, SM1, albumin, and AFP using human (surgical liver resected tissues) and rat liver samples.

For double immunofluorescence examination, the sections were incubated with Protein Block Serum-Free reagent (DAKO, A/S, Denmark) and then incubated with primary antibody overnight at 4 °C. Slides were incubated with FITC- (DAKO, A/S, Denmark) or Alexa Fluor 568-conjugated (Molecular Probes, Eugene, OR) secondary antibodies for 30 minutes at room temperature and then mounted with TO-PRO-3 iodide (Invitrogen, Carlsbad, CA) to label the nuclei. Primary antibodies were as follows: anti-isolectin-B4 (Vector Laboratories, Burlingame, CA) and anti-PCNA (Santa Cruz Biochemistry (SCB), Santa Cruz, CA) antibodies. Four-color imaging was performed in 10 random regions for each sample (z-series, ×63 oil magnification, Zeiss LSM-510 Meta Confocal Microscope; Carl Zeiss, Jena, Germany) and analyzed with Zeiss LSM Image Browser software version 3.5 (Carl Zeiss). The number of PCNA-positive endothelial cells (detected as isolectin-B4^+^ cells) 6 weeks after CCl_4_ injection were morphometrically quantified with the use of all fields of tissue sections (six liver lobules for each of eight rats).

### Western blot analysis

Frozen liver tissues were homogenized in RIPA buffer (Thermo Scientific, Rockford, IL) containing a protein inhibitor cocktail (Sigma, Tokyo, Japan), and the protein concentration was determined with the DC protein assay kit (BIO-RAD Laboratories, Hercules, CA). Proteins were loaded onto NuPAGE 4–12% Bis–Tris gel (Invitrogen), electrophoresed, and electrotransferred to a Fluorotrans Membrane (Pall Life Science, Ann Arbor, MI). Following electrotransfer, the membrane was blocked and incubated overnight at 4 °C with a primary antibody (PCNA, matrix metalloproteinase (MMP)-7, MMP-13, ITG β3 and ITG β5 from SCB; ITG αv from Cell Signaling, Boston, MA; and β-actin, Sigma Chemical). Visualization of the protein signal was achieved with a horseradish peroxidase–conjugated secondary antibody (GE Healthcare UK, Buckinghamshire, UK) and enhanced chemiluminescence western blot analysis system (Amersham Pharmacia Biotech, Piscataway, NJ) using LAS 4000 mini (Fujifilm, Tokyo, Japan) and calculated with the amount of luminescence in each sample using Multigauge software (Fujifilm, Tokyo, Japan).

### Gelatin zymography

For gelatin zymography, precast Novex zymogram gels (10% Tris–Glycine gel with 0.1% gelatin, Invitrogen) were loaded with 50 μg of protein per condition and separated under nonreducing conditions. Gels were placed in 2.5% Triton X-100 solution for 30 minutes and then incubated for 18 hours in enzyme activation buffer (50 mmol/l Tris–HCl, pH = 7.5, with 5 mmol/l CaCl_2_). Gels were then stained with Coomassie blue R-250 and destained in 10% acetic acid and 20% methanol, as described previously.^19,27^

### Statistical analysis

The results were statistically analyzed with the use of a software package (StatView 5.0, Abacus, Concepts, Berkeley, CA). Differences between groups were examined for statistical significance using the Mann–Whitney *U*-test and the Kruskal–Wallis nonparametric analysis of variance. All data are expressed as the mean ± SD, and *P* values less than 5% were considered to indicate significance.

## Figures and Tables

**Table 1:** Expression changes in selected cellular adhesion molecules

*Reference number*	*Gene title*	*Fold change*
NM_000212	Integrin, beta 3 (platelet glycoprotein IIIa, antigen CD61)	263.3226
NM_003005	Selectin P (granule membrane protein 140kDa, antigen CD62)	63.8641
NM_002213	Integrin, beta 5	63.6436
NM_000210	Integrin, alpha 6	31.775
NM_003118	Secreted protein, acidic, cysteine-rich (osteonectin)	16.0977
NM_003638	Integrin, alpha 8	16.0717
NM_002211	Integrin, beta 1 (fibronectin receptor, beta polypeptide, antigen CD29)	15.9935
NM_002203	Integrin, alpha 2 (CD49B, alpha 2 subunit of VLA-2 receptor)	8.0595
NM_002210	Integrin, alpha V (vitronectin receptor, alpha polypeptide, antigen CD51)	8.0387
NM_000211	Integrin, beta 2 (complement component 3 receptor 3 and 4 subunit)	8.0154
NM_002293	Laminin, gamma 1 (formerly LAMB2)	8.0464
NM_003919	Sarcoglycan, epsilon	8.0205
NM_181501	Integrin, alpha 1	4.272
NM_000442	Platelet/endothelial cell adhesion molecule	4.0359
NM_003119	Spastic paraplegia 7 (pure and complicated autosomal recessive)	4.0332
NM_000885	Integrin, alpha 4 (antigen CD49D, alpha 4 subunit of VLA-4 receptor)	3.9976
NM_001331	Catenin (cadherin-associated protein), delta 1	3.9908
NM_001903	Catenin (cadherin-associated protein), alpha 1	3.9846
NM_001904	Catenin (cadherin-associated protein), beta 1	3.9666
NM_000201	Intercellular adhesion molecule 1	2.0138
NM_000610	CD44 molecule (Indian blood group)	2.0122
NM_002209	Integrin, alpha L (antigen CD11A)	2.0071
NM_002291	Laminin, beta 1	2.0066
NM_002205	Integrin, alpha 5 (fibronectin receptor)	2.0004

**Table 2:** RT-PCR primer sequences

*Target gene*	*Annealing temperature*	*Primer*	*Primer sequence*	*Product size (bp)*
hALB	60	Forward	5′-AGA AGG AGA AGC AAA TCA AGA AAC-3′	377
		Reverse	5′-GAA GCA CAG AGA AAA GAG GCA AAA-3′	
hAFP	60	Forward	5′-AGG AAG TAA GCA AAA TGG TGA AAG-3′	367
		Reverse	5′-GTC ATA GCG AGC AGC CCA AAG AAG-3′	
hKRT19	56	Forward	5′-CGA GCA GAA CCG GAA GGA TG-3′	318
		Reverse	5′-AGC CGC TGG TAC TCC TGA TTC-3′	
hACTA2	68	Forward	5′-TCT GGA GGC ACA ACT GGC ATC GT-3′	485
		Reverse	5′-TAC ATA TGT TGT CCC CCT GAT AG-3′	
hVEGF	56	Forward	5′-CAA CAT CAC CAT GCA GAT TAT GC-3′	186
		Reverse	5′-CCA CAG GGA CGG GAT TTC TTG-3′	
hHGF	56	Forward	5′-ACG AAC ACA GCT ATC GGG GTA-3′	282
		Reverse	5′-CAT CAA AGC CCT TGT CGG GAT-3′	
hEGF	56	Forward	5′-GGT CAA TGC AAC CAA CTT CA-3′	383
		Reverse	5′-GGC ATT GAG TAG GTG ATT AG-3′	
hTGFA	66	Forward	5′-CGC CCT GTT CGC TCT GGG TAT-3′	240
		Reverse	5′-AGG AGG TCC GCA TGC TCA CAG-3′	
hFGF2	58	Forward	5′-GGC CAC TTC AAG GAT CCC AAG-3′	399
		Reverse	5′-TCA GCT CTT AGC AGA CA-3′	
hNOS3	63	Forward	5′-GTG ATG GCG AAG CGA GTG AAG-3′	421
		Reverse	5′-CCG AGC CCG AAC ACA CAG AAC-3′	
hANGPT1	59	Forward	5′-GCT GGC AGT ACA ATG ACA GT-3′	339
		Reverse	5′-GTA TCT GGG CCA TCT CCG AC-3′	
hANGPT2	58	Forward	5′-GAG ATC AAG GCC TAC TGT GA-3′	443
		Reverse	5′-AAG TTG GAA GGA CCA CAT GC-3′	
hGAPDH	60	Forward	5′-ACC ACA GTC CAT GCC ATC AC-3′	452
		Reverse	5′-TCC ACC ACC CTG TTG CTG TA-3′	
